# Genomic Structural Equation Modeling Reveals Shared Genetic Architecture and Pleiotropic Hub Genes of Sepsis-Induced Cardiomyopathy

**DOI:** 10.3390/genes17070751

**Published:** 2026-06-30

**Authors:** Min Fang, Bin Zhou, Peng Yu, Xiang Long, Min Shao

**Affiliations:** 1 Department of Critical Care Medicine, The First Clinical Medical College of Anhui Medical University, Hefei 230032, China; 2136020132@stu.ahmu.edu.cn; 2 The Second Clinical Medical College, Nanchang University, Nanchang 330006, China; zhoubin6301@163.com (B.Z.); zhangyuteam2022@163.com (P.Y.)

**Keywords:** sepsis-induced cardiomyopathy, genomic structural equation modeling, shared genetic architecture, multivariate genome-wide association study, AMPK signaling

## Abstract

**Background:** Sepsis-induced cardiomyopathy (SICM) is a life-threatening complication driven by inflammatory cascades. Current genetic studies are restricted to single-trait analyses that cannot capture the shared genetic architecture spanning from immune dysregulation to structural myocardial damage. **Methods:** We applied genomic structural equation modeling to integrate genome-wide association study (GWAS) summary statistics for six phenotypes—sepsis, cardiac troponin I, left ventricular ejection fraction (LVEF), left ventricular diastolic strain rate, right ventricular peak ejection rate, and heart failure—constructing a latent factor for the shared genetic basis of SICM-related phenotypes. Downstream analyses included multivariate GWAS, fine-mapping (SuSiE/FINEMAP), sparse canonical correlation analysis-based transcriptome-wide association study (sCCA-TWAS) with FOCUS prioritization, MAGMA gene-set enrichment, cell-type enrichment (CELLECT), spatial transcriptomic mapping (gsMap), and stratified LD score regression (S-LDSC). **Results:** The model showed adequate fit (CFI = 0.936), with left ventricular diastolic strain rate and LVEF anchoring the factor most strongly (λ = 0.811 and 0.636, respectively). Multivariate GWAS identified 4220 lead variants, of which 4197 did not reach genome-wide significance in any constituent single-trait GWAS. Cross-referencing sCCA-TWAS with FOCUS fine-mapping prioritized 39 genes spanning inflammatory transduction, gap junction remodeling, proteostatic defense, and energy sensing. AMPK signaling was recurrently captured across fine-mapping and transcriptome-wide analyses. CELLECT identified cardiac muscle cells as the sole significant cell type. **Conclusions:** This study provides the first integrative multi-trait genetic framework for the shared genetic basis of SICM-related phenotypes, identifying AMPK as a recurrently captured pleiotropic hub at the inflammation–metabolism intersection and providing a foundation for future biomarker and mechanistic investigations.

## 1. Introduction

Sepsis, defined as life-threatening organ dysfunction caused by a dysregulated host response to infection, is the leading cause of death in intensive care units and confers substantial long-term morbidity among survivors [1]. Among the organ injuries precipitated by sepsis, the heart is one of the most vulnerable. Sepsis-induced cardiomyopathy (SICM)—acute and typically reversible ventricular dysfunction driven by inflammatory cytokine release and mitochondrial dysfunction—has a pooled prevalence of approximately 20% and independently doubles short-term mortality [2]. The severity of myocardial injury during sepsis, however, varies markedly among individuals, pointing to host genetic variation as a contributor to disease progression. Death from infection is strongly heritable, and the first genome-wide association study (GWAS) of sepsis survival identified independent genetic loci associated with prognosis, confirming that common genetic variation shapes sepsis outcomes [3]. Despite this tight biological coupling between sepsis and myocardial injury, genetic investigations have been restricted to single-trait association analyses of either sepsis susceptibility or heart failure (HF) in isolation. This single-trait approach cannot capture the shared genetic architecture that links immune dysregulation, inflammatory mediation, and structural myocardial damage through common pleiotropic pathways.

Large-scale clinical and experimental studies have delineated much of SICM pathophysiology. At the molecular level, sepsis activates NF-κB-mediated pro-inflammatory cascades and triggers cardiomyocyte pyroptosis through Toll-like receptor (TLR) signaling, directly suppressing contractile function [4]. Gene-knockout murine models have demonstrated that TLR4 deficiency markedly attenuates endotoxin-induced myocardial inflammation and cardiac dysfunction, establishing the causal centrality of this pathway [5]. Mitochondrial bioenergetic failure is equally critical. Transcriptomic analysis of myocardium from patients who died of sepsis showed that expression of 198 mitochondrially localized energy production genes—encompassing Krebs cycle and electron transport chain components—declined by an average of 43%, alterations more profound than those observed in end-stage HF [6]. Building upon these molecular insights, candidate gene association studies identified functional polymorphisms in core inflammatory genes, including TNF, TLR4, IL-10, and HMGB1. However, these hypothesis-driven strategies covered only a narrow set of preselected targets, and most failed to replicate in independent cohorts [7]. Although clinical and experimental work have separately characterized inflammatory cascades and mitochondrial energy failure in SICM, recent evidence indicates a complex interplay between these two pathways, with genetic factors implicated [8]. Whether pleiotropic genetic hubs coordinating both inflammatory and metabolic pathways exist remains unknown.

The advent of GWAS has enabled unbiased, genome-wide interrogation of sepsis and cardiovascular disease genetics. Large-scale meta-analyses identified landmark loci for sepsis survival, including common variants in FER that reduced 28-day mortality in pneumonia-related sepsis by 44% [9]. A GWAS of sepsis-associated acute respiratory distress syndrome revealed a significant contribution of variants in FLT1, encoding vascular endothelial growth factor receptor 1 (VEGFR-1), with functional experiments validating its gene-regulatory role [10]. These studies, however, examined single phenotypes in isolation and could not determine whether identified loci influence sepsis and cardiac injury through shared or independent genetic pathways. Mendelian randomization (MR) studies have advanced statistical associations toward causal inference, revealing a causal relationship between HF and sepsis risk mediated by nerve growth factor, and identifying serum ferritin as a shared risk factor for both conditions [11]. These paired-exposure analyses, however, tested one exposure against one outcome at a time, relying on general cardiomyopathy as a genetic proxy in the absence of dedicated GWAS data for acute SICM [12]. This single-exposure versus single-outcome framework cannot model the multi-pathway synergistic damage that characterizes SICM, in which inflammatory, metabolic, and structural injury dimensions operate simultaneously. No study has yet integrated multiple interconnected phenotypes spanning systemic infection, inflammatory mediation, and myocardial injury to construct the shared genetic basis of SICM-related phenotypes covering the full pathological cascade.

To address these gaps, this study applies genomic structural equation modeling (SEM)—a multivariate statistical genetic framework that models the factor structure underlying genetic correlations among traits and identifies variants with effects on latent genetic factors—to characterize the shared genetic basis of SICM-related phenotypes by integrating large-scale GWAS summary statistics. Six phenotypes were selected to span the full SICM pathological cascade, from systemic infection (sepsis) and cardiomyocyte injury (circulating cardiac troponin I [cTnI]) through biventricular functional impairment (left ventricular ejection fraction [LVEF], left ventricular diastolic strain rate, and right ventricular peak ejection rate) to clinical HF. Because no large-scale GWAS of directly diagnosed SICM is currently available, this latent factor was constructed from these six proxy phenotypes and should be understood as approximating, rather than fully representing, SICM itself. Downstream analyses encompassed multivariate GWAS, statistical fine-mapping, cross-validated transcriptomic association, gene-set and cell-type enrichment, and spatial transcriptomic mapping, together providing an integrative framework for the shared genetic basis of SICM-related phenotypes and a foundation for biomarker discovery and therapeutic target identification.

## 2. Materials and Methods

### 2.1. Data Sources and Variant-Level Quality Filtering

Six phenotypes were selected to span the full pathological cascade of SICM, from systemic infection through myocardial cellular injury and biventricular functional impairment to clinical HF. Sepsis (11,643 cases, 474,841 controls) represented the initiating insult, and circulating cTnI (N = 27,254) served as a direct indicator of cardiomyocyte injury. Three cardiac magnetic resonance imaging-derived measures characterized distinct dimensions of ventricular performance, including LVEF (N = 56,210), left ventricular diastolic strain rate (N = 23,321), and right ventricular peak ejection rate (N = 35,928). HF (14,262 cases, 471,898 controls) served as the terminal clinical endpoint. All summary statistics were derived from European-ancestry cohorts. Data sources and sample sizes are detailed in Table S1. Autosomal variants were filtered against the 1000 Genomes Project Phase 3 European reference panel. Single-nucleotide polymorphisms (SNPs) were removed if they had a minor allele frequency (MAF) below 0.01, carried ambiguous allelic coding, or mapped within the major histocompatibility complex (MHC) region on chromosome 6 (25–35 Mb).

### 2.2. Latent Factor Modeling via Genomic SEM

Genomic SEM models the factor structure underlying genetic correlations among multiple traits and identifies variants whose effects operate through shared latent genetic dimensions rather than any single constituent phenotype. The Genomic SEM R package (version 0.0.5) was used to model the latent genetic architecture shared across the six SICM-related traits in a two-stage framework. In the first stage, quality-controlled summary statistics were restricted to HapMap3 SNPs, and multivariate linkage disequilibrium score regression (LDSC) (v1.0.1) estimated the genetic covariance matrix and its associated sampling covariance matrix. In the second stage, all autosomal SNPs passing the quality-control filters described above were retained for the multivariate GWAS. A common-factor confirmatory model was fitted under the assumption that a single latent genetic factor drives shared susceptibility across the six traits. Hereafter, we refer to this latent factor as the SICM-related latent factor, reflecting that it captures the shared genetic basis underlying the diagnostic dimensions of SICM rather than SICM as a discretely diagnosed entity. The single-factor model was adopted in the interest of parsimony and interpretability. As a sensitivity analysis, a correlated two-factor model, with phenotypes assigned to factors on the basis of an exploratory factor analysis, was additionally specified and compared against the single-factor model. Model fit was evaluated using the comparative fit index (CFI), standardized root mean square residual (SRMR), model chi-square (χ^2^), and Akaike information criterion (AIC). To assess potential inflation of the factor-level association statistics, we computed the genomic inflation factor and the LDSC intercept. The LDSC intercept of each input GWAS was also examined to distinguish population stratification from sample overlap. Note that Genomic SEM is unbiased in the presence of varying and unknown sample overlap across the contributing GWAS, as the cross-trait intercepts estimated via multivariate LDSC are used to estimate and account for sample overlap. Per-variant heterogeneity was evaluated using the Cochran’s Q statistic (QSNP), which tests whether a variant’s effects across the six indicator traits are fully consistent with mediation through the shared latent factor. Variants showing significant heterogeneity (QSNP *p* < 0.05) were excluded to ensure that the retained factor-level associations reflect effects operating through the shared genetic dimension.

### 2.3. Genome-Wide Locus Discovery and Detection Gain Assessment

Expanding the latent factor model to incorporate individual variants, genomic regions associated with the SICM-related latent factor were annotated using the Functional Mapping and Annotation (FUMA) platform (v1.3.7) [13]. Genome-wide significant SNPs (*p* < 5 × 10^−8^) were identified, and lead variants were defined by pairwise linkage disequilibrium thresholds (r^2^ < 0.1). To quantify the detection gain of the multivariate approach, loci were classified as additionally detected when their lead SNP was located more than 1 Mb from any genome-wide significant variant (*p* < 5 × 10^−8^) in any of the six constituent single-trait GWAS. Cross-referencing against the GWAS Catalog [14] provided independent verification. Gene-based and gene-set association tests were performed using MAGMA (v1.08) [15], with significance determined at FDR < 0.05. To refine association signals to putative causal variants, statistical fine-mapping was performed using SuSiE and FINEMAP [16] implemented in the echolocatoR R package (version 2.0.3). A ±250 kb window flanking each lead SNP defined the analytical region for posterior probability (PP) computation. Variants with a mean PP exceeding 0.95 within 95% credible sets were prioritized as candidate causal variants.

### 2.4. Transcriptome-Wide Association and Pathway Enrichment

To bridge genetic associations to gene expression regulation, transcriptome-wide association analysis was performed using the sparse canonical correlation analysis-based transcriptome-wide association study (sCCA-TWAS) framework [17]. Pre-computed expression quantitative trait locus (eQTL) weights from the Genotype-Tissue Expression (GTEx) project version 8, accessed through the TWAS Atlas database [18], provided the reference expression panel. Genes reaching FDR < 0.05 in the sCCA-TWAS analysis were subsequently fine-mapped using Fine-mapping Of CaUsal gene Sets (FOCUS), a downstream Bayesian procedure that refines transcriptome-wide association signals [19], which estimates a posterior inclusion probability (PIP) for each candidate gene by jointly modeling summary statistics and eQTL weights while accounting for linkage disequilibrium and potential colocalization among neighboring genes. Genes with PIP > 0.8 were designated as high-confidence candidate genes. As part of the sCCA-TWAS analysis, colocalization between expression and factor-level association signals was assessed at each gene by estimating the posterior probability of a shared causal variant (PP4). Pathway-level annotation was performed using gene-set enrichment analysis (GSEA) with the Molecular Signatures Database (MSigDB). The input gene set comprised genes prioritized by MAGMA from the latent factor analysis. All protein-coding genes served as the background set, and enrichment significance was evaluated at FDR < 0.05.

### 2.5. Cell-Type Enrichment and Spatial Transcriptomic Mapping

Briefly, cell-type enrichment analysis was performed using CELLECT [20], which integrates GWAS summary statistics with single-cell RNA sequencing (scRNA-seq) expression profiles from the Tabula Muris mouse atlas [21] to test whether the h^2^SNP of the SICM-related latent factor was preferentially concentrated in genes with cell-type-restricted expression. Within CELLECT, cell-type-specific expression profiles were first preprocessed by CELLEX and then combined with stratified LDSC (S-LDSC) for the enrichment test. In a parallel analysis, S-LDSC with the baseline-LD model [22] partitioned h^2^SNP across functional genomic annotation categories to quantify the relative contribution of each category. Statistical significance for both analyses was set at FDR < 0.05. Spatial localization of genetic signals associated with the SICM-related latent factor within the developmental tissue context was performed using genetically informed spatial mapping (gsMap, v1.73.5). This analysis integrated latent factor GWAS summary statistics with mouse embryonic spatial transcriptomics data (E16.5_E1S1.MOSTA), with cross-species gene mapping facilitated by a homologue file linking mouse and human orthologous genes.

## 3. Results

### 3.1. Genetic Correlations and SNP-Based Heritability

**Figure 1:**
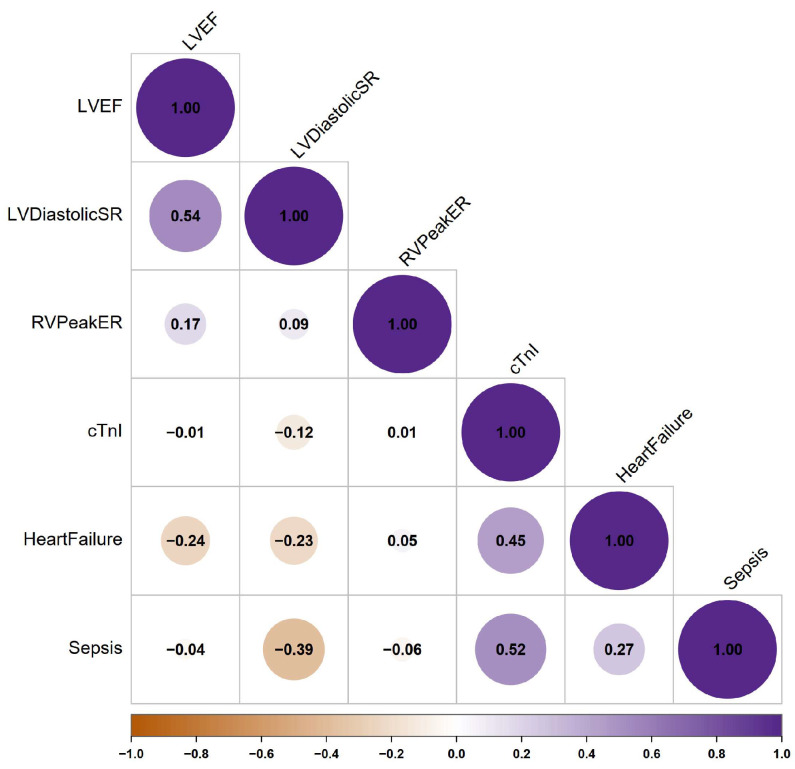
Pairwise genetic correlations among six SICM-related traits estimated by LDSC.

LDSC analysis revealed variable h^2^SNP across the six SICM-related traits (Table S2). Left ventricular diastolic strain rate (h^2^SNP = 0.184, Z = 8.00) and LVEF (h^2^SNP = 0.151, Z = 7.13) exhibited the highest estimates, followed by right ventricular peak ejection rate (h^2^SNP = 0.106, Z = 7.47) and HF (h^2^SNP = 0.077, Z = 8.32). cTnI (h^2^SNP = 0.034, Z = 1.88) and sepsis (h^2^SNP = 0.029, Z = 2.52) displayed lower heritability. The pairwise genetic correlation matrix revealed biologically coherent clustering (Table S3, Figure 1). The two left ventricular functional measures were strongly positively correlated (rg = 0.535, SE = 0.081) and both exhibited negative genetic correlations with HF (rg = −0.241 and −0.230, respectively), as expected given that higher LVEF and strain rate indicate better function, whereas HF represents its failure. cTnI showed moderate positive genetic correlations with both HF (rg = 0.446, SE = 0.174) and sepsis (rg = 0.518, SE = 0.297). HF and sepsis were positively correlated at the genetic level (rg = 0.268, SE = 0.162).

### 3.2. Latent Factor Model and Factor Structure

A common-factor confirmatory model fitted to the genetic covariance matrix of all six traits did not reject adequate fit (χ^2^ = 16.07, df = 9, *p* = 0.065, AIC = 40.07, CFI = 0.936, Table S4). As a sensitivity analysis, a data-driven correlated two-factor model was also evaluated. Although it showed a modest improvement in fit (χ^2^ = 10.79, CFI = 0.975, AIC = 36.79), the two factors were substantially negatively correlated (rg = −0.44), indicating two poles of a single underlying axis rather than distinct genetic dimensions. The single-factor model was therefore retained on the grounds of parsimony and interpretability. Standardized factor loadings indicated that the SICM-related latent factor was most strongly anchored by left ventricular diastolic strain rate (λ = 0.811) and LVEF (λ = 0.636) (Table S5). Sepsis (λ = −0.424) and HF (λ = −0.344) loaded negatively, consistent with the directional expectation that higher latent factor values correspond to better cardiac function and lower disease burden. Right ventricular peak ejection rate (λ = 0.134) and cTnI (λ = −0.204) contributed modestly. The genomic inflation factor of the factor-level statistics was 1.32. The LDSC intercept was 1.30 (SE = 0.012), whereas the LDSC intercept of each of the six input GWAS was close to 1 (range 0.99 to 1.03, Table S2), indicating that the elevated factor-level intercept reflects partial sample overlap among the input GWAS rather than population stratification. The quantile-quantile plot (Figure S1) showed early lift-off followed by a smooth departure from the null in the tail, consistent with polygenic architecture.

### 3.3. Genome-Wide Locus Discovery and Detection Gain

**Figure 2:**
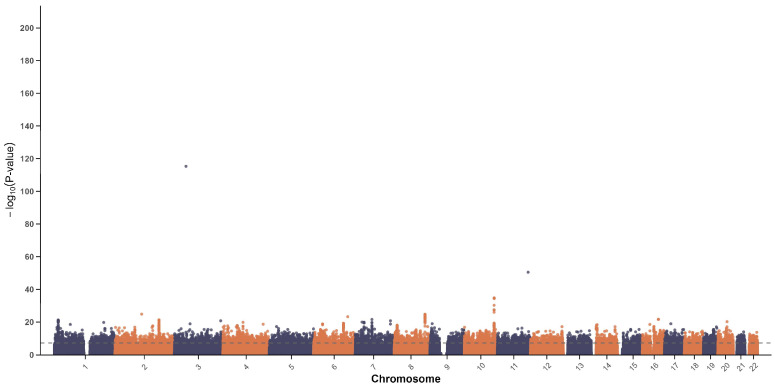
Manhattan plot of the multivariate GWAS for the SICM-related latent factor. Each dot represents a single-nucleotide polymorphism, with dark blue and orange indicating alternating chromosomes. Magenta dots denote the lead variants that overlap previously reported cardiovascular loci (rs1763604, rs16866400, rs4151702, rs12541595, and rs17617337). The grey dashed horizontal line marks the genome-wide significance threshold (*p* = 5 × 10^−8^).

The Genomic SEM-based GWAS generated association statistics for 6,903,960 autosomal SNPs. FUMA identified 16,409 genome-wide significant SNPs (*p* < 5 × 10^−8^, Figure 2, Table S6), consolidated into 4220 lead variants by pairwise LD thresholds (r^2^ < 0.1, Table S7). Of these, 4197 did not reach genome-wide significance in any of the six constituent single-trait GWAS (Table S8), consistent with the detection gain expected from the multivariate approach. As these associations have not been replicated in an independent cohort, they require validation in future studies. Among the variants overlapping established cardiovascular GWAS loci (Table S9), several mapped to regions with well-characterized cardiovascular associations. rs17617337 on chromosome 10 (*p* = 1.82 × 10^−35^) mapped to a region previously associated with LVEF, hypertrophic cardiomyopathy, dilated cardiomyopathy, and high-sensitivity cardiac troponin T levels. rs12541595 on chromosome 8 (*p* = 1.68 × 10^−25^) mapped to a locus associated with atrial fibrillation, left ventricular structure, and hematological traits. rs16866400 (chr2, *p* = 4.24 × 10^−22^) has been linked to QT interval and atrial fibrillation, and rs1763604 (chr1, *p* = 5.15 × 10^−22^) to a locus associated with hypertrophic and dilated cardiomyopathy. rs4151702 (chr6, *p* = 1.41 × 10^−19^) mapped to a locus spanning coronary artery disease, HF, and immune-related traits including systemic lupus erythematosus and rheumatoid arthritis, suggesting a convergence of cardiovascular and immune genetic signals within the SICM-related latent factor. The functional annotation profile of significant variants was broadly similar to the genomic background, with the majority falling in intronic (37.0%) and intergenic (45.1%) regions. Notably, ncRNA exonic variants were enriched (1.28-fold, *p* = 2.24 × 10^−9^), as were variants in upstream regulatory regions (1.22-fold, *p* = 1.71 × 10^−6^) and 3′ UTR sequences (1.15-fold, *p* = 0.002), suggesting regulatory noncoding elements as primary mediators. Exonic variants were underrepresented (0.80-fold, *p* = 3.45 × 10^−6^).

### 3.4. Fine-Mapping of Causal Variants

**Figure 3:**
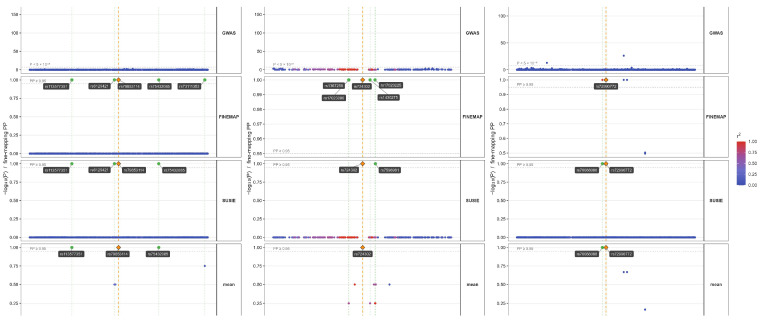
Fine-mapping of the representative locus with the highest posterior probability. Within each column, the four panels show, from top to bottom, the GWAS −log_10_(P) and the fine-mapping posterior probability (PP) from FINEMAP, SuSiE, and their mean. Each dot represents a single-nucleotide polymorphism, colored by linkage disequilibrium r^2^ to the lead SNP, ranging from blue (low) to red (high). The orange diamond marks the lead SNP, and green dots denote SNPs in the 95% credible set. The horizontal dotted lines mark the genome-wide significance threshold (*p* < 5 × 10^−8^) in the GWAS panels and the PP ≥ 0.95 threshold in the fine-mapping panels, as labeled within each panel. The orange dashed vertical lines indicate the positions of the lead SNPs, and the green dashed vertical lines indicate the positions of SNPs in the 95% credible sets.

Of the 16,409 genome-wide significant SNPs, statistical fine-mapping using SuSiE and FINEMAP resolved 3128 variants into 95% credible sets (Table S10). Three variants achieved the highest PP (mean PP = 1.0) including rs79853114 mapping to RP5-1031J8.1 on chromosome 20 (*p* < 1 × 10^−200^), rs724302 mapping to AC138623.1 on chromosome 2 (*p* < 1 × 10^−200^), and rs72990772 mapping to R3HDM2P2 on chromosome 6 (*p* = 1.35 × 10^−137^). The fine-mapping result for rs724302 is shown in Figure 3 as a representative example, with full results for all loci in Table S10.

### 3.5. Transcriptome-Wide Association and Causal Gene Prioritization

**Figure 4:**
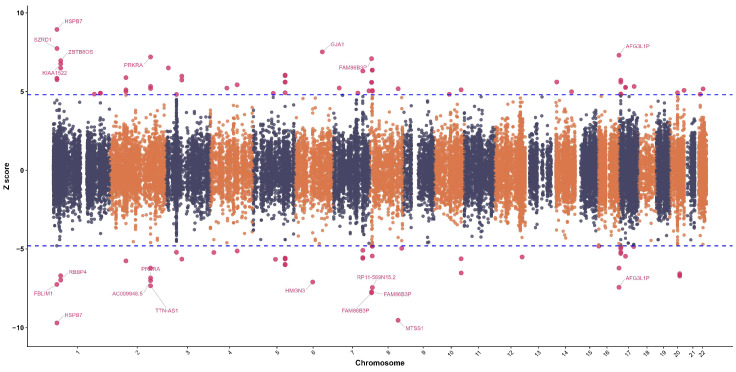
Transcriptome-wide association Z-scores for the SICM-related latent factor across chromosomes.

sCCA-TWAS identified 97 genes at FDR < 0.05, of which 53 had positive and 44 had negative TWAS Z-scores (Figure 4). FOCUS nominated 163 genes with PIP exceeding 0.8. Cross-referencing the two steps yielded 39 genes that were both associated at the transcriptome-wide level and supported by FOCUS fine-mapping (Table 1), which we prioritized for further investigation. Among these, HSPB7 on chromosome 1 exhibited the strongest signal across multiple sCCA features (TWAS Z = −9.70 and 8.95; FDR *p* = 2.98 × 10^−22^ and 3.62 × 10^−19^; PIP = 1.0 in both). MTSS1 on chromosome 8 (TWAS Z = −9.53, FDR *p* = 1.58 × 10^−21^, PIP = 1.0), AFG3L1P on chromosome 16 (TWAS Z = −7.44, FDR *p* = 1.04 × 10^−13^, PIP = 1.0), and GJA1 on chromosome 6, encoding connexin 43 (Cx43; TWAS Z = 7.52, FDR *p* = 5.28 × 10^−14^, PIP = 1.0), followed. Additional genes with PIP = 1.0 included BAG3, TTN-AS1, PDZRN3, FAM167B, MCMBP, SLC6A6, SERINC3, PRKRA, and CTD-2260A17.2. Genes encoding key signaling molecules also reached high-confidence status including PRKCA (PIP = 0.999), VTN (PIP = 0.999), PRKAB1 (PIP = 0.999), TNFAIP1 (PIP = 0.996), and RASA1 (PIP = 0.997). Colocalization analysis supported a shared causal variant (PP4 > 0.75) for many of the prioritized genes, including PRKAB1 (0.811), GJA1 (0.99), HSPB7 (0.992), SERINC3 and AFG3L1P (1.0) (Table 1). Colocalization was treated as complementary evidence. Because PP4 tests the single-shared-variant hypothesis, genes with strong transcriptome-wide and fine-mapping support but limited colocalization were retained.

### 3.6. MAGMA Gene-Based Analysis and Gene-Set Enrichment

**Figure 5:**
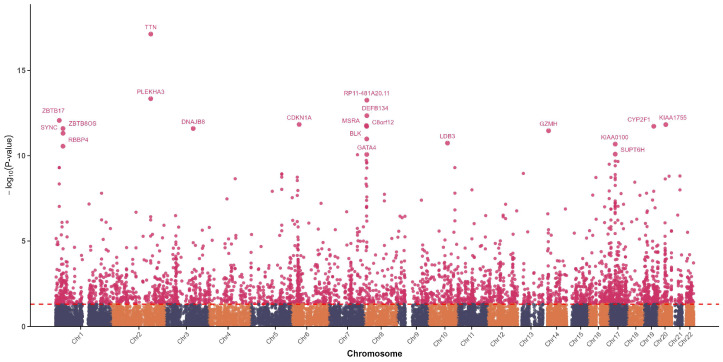
MAGMA gene-based association results for the SICM-related latent factor. Each dot represents a gene, with dark blue and orange indicating alternating chromosomes. Magenta dots denote genes surpassing the significance threshold, and the top genes are labeled. The red dashed horizontal line marks the significance threshold (FDR < 0.05).

MAGMA gene-based analysis identified 1029 genes associated with the SICM-related latent factor at FDR < 0.05 (Table S11, Figure 5). The strongest association was observed for TTN (Z = 8.53, *p* = 7.46 × 10^−18^), encoding titin, a gene central to cardiac structural integrity and contractile reserve, followed by PLEKHA3 (Z = 7.45, *p* = 4.55 × 10^−14^) on chromosome 2. Gene-set enrichment analysis returned six enriched sets at FDR < 0.05 (Table S12), comprising non-specific GWAS Catalog sets such as remission after selective serotonin reuptake inhibitor treatment, blood pressure response to alcohol consumption, joint mobility, and dietary consumption patterns. As these sets are non-specific and do not define clear inflammatory or metabolic pathways, they were treated as exploratory and were not interpreted as defining specific biological pathways.

### 3.7. Cell-Type Enrichment, Spatial Transcriptomic Mapping, and Functional Annotation

CELLECT identified cardiac muscle cells as the sole cell type with significant heritability enrichment for the SICM-related latent factor (coefficient = 4.37 × 10^−9^, FDR *p* = 0.048) (Table S13). The gsMap localized genetic signals associated with the SICM-related latent factor across 21 tissue annotations in the E16.5 mouse embryonic atlas (Table S14). The strongest enrichment was observed in lung (*p* = 2.16 × 10^−8^), followed by muscle (*p* = 2.02 × 10^−7^), connective tissue (*p* = 2.87 × 10^−7^), and kidney (*p* = 9.73 × 10^−7^). Cartilage primordium (*p* = 1.06 × 10^−5^), smooth muscle (*p* = 1.26 × 10^−4^), brain (*p* = 1.26 × 10^−4^), and heart (*p* = 2.36 × 10^−3^) also reached significance, as did adrenal gland (*p* = 4.74 × 10^−4^), epidermis (*p* = 5.17 × 10^−4^), and jaw and tooth (*p* = 1.22 × 10^−3^). S-LDSC partitioning revealed pronounced enrichment in coding regions (50.7-fold), evolutionarily conserved sequences (49.1-fold), and H3K9 acetylation marks (14.3-fold, FDR *p* = 1.49 × 10^−4^) (Table S15). DNase I hypersensitive sites (15.0-fold), fetal DNase I hypersensitive regions (14.7-fold), H3K27 acetylation marks (6.4-fold), and H3K4me3 marks at promoters (5.0-fold) were also enriched.

## 4. Discussion

This study applied Genomic SEM to six SICM-related phenotypes and identified a single latent genetic factor with adequate model fit, supporting the existence of a shared genetic dimension that spans the SICM-related phenotypes from systemic infection through myocardial cellular damage to clinical HF. Before discussing these findings, we note an important interpretive boundary. To our knowledge, no large-scale GWAS of SICM is currently available. The latent factor analyzed here was therefore constructed as a multi-trait genetic proxy spanning the diagnostic dimensions of SICM. Accordingly, the findings below should be read as characterizing the shared genetic basis of SICM-related phenotypes, rather than the genetic architecture of SICM as a directly diagnosed entity. The multivariate approach detected 4220 lead variants, of which 4197 did not reach genome-wide significance in any constituent single-trait GWAS. We refer to these as detected at the latent-factor level rather than as novel, as the lack of detection in the single-trait analyses does not by itself establish novelty and these associations await independent replication. Instead of the candidate inflammatory genes investigated in prior hypothesis-driven studies (TNF, TLR4, IL-10, HMGB1), fine-mapping, sCCA-TWAS, and MAGMA converged on a distinct set of genes bridging innate immune sensing (NOD2), vascular permeability regulation (VEGFA), and mitochondrial energy metabolism (CPT1A) to inflammatory signal transduction (TNFAIP1), gap junction remodeling (GJA1), cardiac proteostatic defense (HSPB7, BAG3), and energy metabolic sensing (PRKAB1). Cell-type analysis identified cardiac muscle cells as the sole significant cell type. AMPK signaling was recurrently captured across multiple analytical tiers, emerging as a pleiotropic hub coordinating the inflammation–metabolism intersection.

The involvement of innate immune sensing, vascular, and metabolic genes among the highest-confidence fine-mapped variants is consistent with the multi-pathway nature of SICM-related phenotypes and points to previously unrecognized genetic links between these pathways. NOD2 encodes nucleotide-binding oligomerization domain-containing protein 2, an intracellular pattern recognition receptor that senses bacterial muramyl dipeptide and activates innate immune responses through NF-κB. A systematic review confirmed NOD2 as a shared genetic risk gene for both sepsis and cardiovascular disease, with the adverse allele exerting concordant effects in both conditions [23]—our identification of NOD2 at the latent-factor level aligns with and extends this observation to the multi-trait architecture across SICM-related phenotypes. In cardiac tissue, ischemia–reperfusion upregulates NOD2 expression, and ligand activation induces cardiomyocyte apoptosis and inflammation through JNK, p38 MAPK, and NF-κB [24]. Beyond inflammatory signaling, NOD2 activation suppresses mitochondrial autophagy through the NOD2/AMPK/LC3 pathway, decreasing mitochondrial membrane potential and increasing reactive oxygen species production [25]. NOD2 variants may therefore influence susceptibility to SICM-related phenotypes by simultaneously modulating NF-κB-mediated inflammation and AMPK-dependent mitochondrial quality control. Fine-mapping also highlighted vascular permeability regulation as a contributor to the genetic risk shared across SICM-related phenotypes. VEGFA encodes vascular endothelial growth factor A, a central regulator of endothelial barrier function. Blocking VEGF signaling with soluble Flt-1 improved cardiac function and survival in sepsis models [26], and endotoxemia induced transcriptional upregulation of VEGF in the left ventricle, accompanied by increased microvascular heterogeneity and decreased contractility [27]. An independent MRSA sepsis model confirmed that increased myocardial VEGF mRNA expression paralleled vascular hyperpermeability and cardiovascular collapse [28]. A previous GWAS identified significant associations between FLT1 (encoding VEGFR-1) variants and sepsis-associated acute respiratory distress syndrome [10]. The present study independently identified multiple high-confidence variants in its ligand VEGFA. This ligand–receptor genetic convergence supports a central role for the VEGF pathway in sepsis-related multi-organ injury. In addition to inflammatory and vascular pathways, fine-mapping implicated mitochondrial energy metabolism through CPT1A, which encodes carnitine palmitoyltransferase 1A, the rate-limiting enzyme catalyzing long-chain fatty acid entry into the mitochondrial matrix for β-oxidation—the primary source of cardiac ATP [29]. CPT1A has been recognized as a central node in cardiac metabolic flexibility [30], and in LPS-induced sepsis models, its expression declined alongside fatty acid β-oxidation derangement and cardiac injury, while therapeutic restoration of CPT1A ameliorated multi-organ damage [31]. Transcriptomic analysis of myocardium from sepsis decedents revealed an average 43% decline in 198 mitochondrial energy genes [6], providing an independent transcriptome-level context for the CPT1A genetic signal identified here. The convergence of these fine-mapping results on innate immune sensing, vascular permeability regulation, and mitochondrial energy metabolism illustrates the multi-pathway nature of the shared genetic basis across SICM-related phenotypes.

Having established the variant-level architecture through fine-mapping, TWAS combined with FOCUS and MAGMA prioritized convergent genes whose transcriptional effects span inflammatory execution, electrical conduction, proteostatic defense, and energy sensing. TNFAIP1 was originally identified as an immediate-early response gene of TNF-α in endothelial cells, with transcriptional activation driven by Sp1 [32]. Functionally, TNFAIP1 acts as an adaptor protein of the Cullin3 E3 ubiquitin ligase mediating RhoB degradation. Its downregulation blocks this degradation and induces IL-6 and IL-8 secretion under TNF-α stimulation via p38/JNK MAPK activation [33]. In myocardial ischemia–reperfusion models, silencing TNFAIP1 alleviated cardiomyocyte apoptosis, oxidative stress, and inflammation through Akt/GSK-3β/Nrf2 signaling [34]. If NOD2 represents the upstream sensor initiating NF-κB-mediated cascades, TNFAIP1 represents their downstream execution against cardiomyocytes—the shared architecture across SICM-related phenotypes thus encompasses the full inflammatory arc from sensing to effector damage. GJA1 shifts the focus from inflammatory injury to its functional cardiac consequences. GJA1 encodes Cx43, the principal gap junction protein responsible for synchronous electrical conduction between cardiomyocytes. In a sepsis-induced myocardial dysfunction model, LPS administration reduced ejection fraction and fractional shortening, accompanied by disorganization, fragmentation, and lateralization of Cx43—changes closely associated with p38-mediated Cx43 serine-368 phosphorylation [35]. Cx43 downregulation combined with PI3K/AKT activation attenuated LPS-induced myocardial apoptosis [36], and altered Cx43 expression under sepsis led to electrical remodeling and increased ventricular fibrillation susceptibility [37]. GJA1 variants may therefore influence SICM susceptibility by disrupting cardiomyocyte electrical coupling and promoting arrhythmogenesis. The simultaneous identification of HSPB7 and BAG3 revealed the genetic basis of cardiomyocyte-intrinsic defense within the shared architecture across SICM-related phenotypes. HSPB7 encodes a cardiac-specific small heat shock protein that maintains normal actin thin filament arrangement within sarcomeres. Cardiomyocyte-specific knockout was embryonically lethal, with abnormal actin bundles forming within sarcomeres [38]. Within the cardiac small heat shock protein family, HSPB5, HSPB6, and HSPB8 mediate chaperone functions through the co-chaperone BAG3, while HSPB7 performs BAG3-independent functions—the two arms constitute complementary pathways for cardiac proteostatic maintenance [39]. BAG3 extends beyond protein quality control. It preferentially localizes to mitochondria in cardiac cells, and its downregulation reduces mitophagy and enhances fibroblast activation [40]. BAG3’s mitochondrial role bridges proteostatic defense to the mitochondrial dimension established through CPT1A in the preceding section. PRKAB1 connects inflammatory regulation to energy metabolism, completing the biological circuit. PRKAB1 encodes the β1 regulatory subunit of AMPK, the central cellular energy sensor activated upon declining ATP/AMP ratios. In sepsis-induced myocardial injury models, AMPK levels declined following LPS treatment alongside mitochondrial dysfunction, while SirT3-mediated AMPK activation improved mitochondrial biogenesis and maintained redox balance [41]. The indispensability of AMPK in septic cardioprotection has been confirmed through dual validation using AMPK-deficient mice and pharmacological inhibitors [42]. AMPK phosphorylation also inhibits Drp1-mediated mitochondrial fission and suppresses NLRP3 inflammasome–mediated cardiomyocyte pyroptosis [43], revealing a dual hub bridging energy metabolism and inflammatory regulation. This convergence pattern is striking: NOD2 regulates mitophagy through the AMPK/LC3 pathway, CPT1A is regulated by AMPK phosphorylation, BAG3’s mitophagy function is linked to AMPK signaling, and PRKAB1 encodes the AMPK subunit itself. The same signaling hub was captured across fine-mapping, transcriptomic association, and gene-region cumulative association—spanning immune sensing, mitochondrial quality control, fatty acid oxidation, and proteostasis. We are not claiming that AMPK directly mediates all pathological dimensions of SICM. indeed, several identified genes (GJA1, HSPB7) operate through AMPK-independent mechanisms. Instead, we suggest that AMPK signaling represents the most frequently recaptured node across multiple analytical tiers, making it a pleiotropic integrative target rather than the sole causal pathway. The genetic prioritization of AMPK signaling and its linked candidates (PRKAB1, NOD2, CPT1A, BAG3) provides a focused basis for future functional validation. Targeted gene-perturbation and AMPK-modulation experiments in cellular and animal models of SICM would directly test the integrative role proposed here.

At the cellular level, CELLECT identified cardiac muscle cells as the sole cell type reaching significance, directly implicating cardiomyocytes rather than immune or endothelial cells as the primary cellular effectors of the shared genetic liability across SICM-related phenotypes. This is consistent with the cardiomyocyte-specific effectors described above—HSPB7 and BAG3 in particular. A recent single-nucleus RNA sequencing study revealed that contractile cardiomyocytes converted into an injury-responsive subtype during early sepsis, driven by ERRγ downregulation—reducing contractility but protecting against irreversible damage by suppressing reactive oxygen species [44]. Although immune cells did not reach significance in CELLECT, this does not preclude their role in SICM. Cardiac resident TREM2hi macrophages maintain cardiomyocyte homeostasis by scavenging dysfunctional mitochondria, and their functional deficiency leads to damaged mitochondrial accumulation and cardiac dysfunction [45]. Immune cell contributions to SICM may therefore operate primarily in service of cardiomyocyte mitochondrial homeostasis rather than as independent heritability carriers. Spatial transcriptomic mapping revealed the multi-tissue distribution of genetic signals associated with the SICM-related latent factor. CELLECT measures heritability concentration within defined cell types, whereas gsMap captures the developmental spatial breadth of expression. That these two approaches yield complementary rather than contradictory signals is consistent with shared molecular mechanisms—particularly mitochondrial dysfunction—operating across heart, lung, and kidney. The biological plausibility of lung ranking first is supported by experimental evidence that AQP9 inhibition simultaneously attenuated cardiac, renal, and pulmonary injury in polymicrobial sepsis [46], and genetic deletion of PPIF alleviated myocardial dysfunction while concurrently reducing lung and liver injury [47]. S-LDSC heritability partitioning revealed pronounced enrichment in coding regions and evolutionarily conserved sequences, indicating that SICM-driving variants concentrate in functional elements under strong evolutionary constraint. Enrichment of H3K9 acetylation, DNase I hypersensitive sites, and promoter-associated H3K4me3 marks pointed to active transcription and open chromatin regulatory elements, indicating that the genetic regulation of SICM-related phenotypes operates predominantly through modulating gene expression—a finding methodologically concordant with the TWAS-based causal gene identification strategy employed in this study.

Several limitations should be considered when interpreting these findings. First and most fundamentally, no GWAS of directly diagnosed SICM is currently available, so the latent factor approximates rather than fully represents SICM and may partly overlap with general cardiovascular architecture. Validation against future genetic datasets of echocardiographically verified SICM, for example ICU cohorts with reversible left ventricular dysfunction in sepsis, whether through direct GWAS comparison or through genetic correlation and polygenic-score analyses against the present latent factor, represents the most important direction for future work. Second, because the analysis is based on summary statistics and does not condition on pre-existing heart failure or cardiomyopathy, signals at established cardiac-disease genes such as TTN cannot be fully distinguished between susceptibility acting through the septic-stress response and general cardiac-disease susceptibility that is also relevant under sepsis. Although baseline cardiac reserve is itself a determinant of SICM, separating these components would require GWAS of directly diagnosed SICM with conditioning on pre-existing cardiac disease. Third, partial sample overlap among the input GWAS may contribute to the elevated factor-level LDSC intercept, so the precise number of lead variants should be interpreted with caution. Fourth, LVEF is a ratio trait, defined as stroke volume divided by end-diastolic volume. Genetic associations with ratio phenotypes are not unique, as in sufficiently large samples, a variant associated with either the numerator or the denominator is expected to associate with the ratio. Its contribution to the latent factor should therefore be interpreted with this caveat, and sensitivity analyses partitioning the ratio into its stroke-volume and end-diastolic-volume components represent a direction for future work [48,49]. Fifth, the gene-set enrichment returned non-specific sets that weaken its pathway-level interpretability and likely reflect residual noise or pleiotropy, as the constituent traits, particularly sepsis and heart failure, have documented genetic correlations with metabolic, neuropsychiatric, and lifestyle phenotypes. The pathway-level interpretation therefore rests on the functionally specific genes prioritized by fine-mapping and transcriptome-wide analysis rather than on the gene-set enrichment. Sixth, the colocalization analysis was based on GTEx expression panels derived from non-septic tissue, as eQTL data from human sepsis-affected cardiac tissue are not currently available. Colocalization in sepsis-specific tissue, as such resources emerge, would further strengthen the causal link to SICM specifically. Seventh, the gsMap analysis relied on a mouse embryonic spatial atlas mapped to human genes through homologue conversion, and its tissue ranking should therefore be interpreted with caution rather than as a direct measure of organ involvement in adult SICM. Finally, beyond the targeted gene-perturbation experiments motivated above, network-level perturbation combined with phosphoproteomic or metabolomic profiling would directly test the proposed inflammation–metabolism–proteostasis axis, and the present genetic findings provide a defined set of candidates and pathway relationships to guide such experiments.

## 5. Conclusions

This study applied Genomic SEM to construct the first integrative multi-trait framework for the shared genetic basis of SICM-related phenotypes, integrating six phenotypes spanning from systemic infection through myocardial cellular injury to clinical HF. The multivariate approach detected many variants that did not reach genome-wide significance in the contributing single-trait GWAS, consistent with the expected detection gain of the multivariate framework. These associations have not been replicated in an independent cohort and require validation in future studies. Fine-mapping and gene-level prioritization converged on several biological themes including innate immune sensing and NF-κB-mediated inflammatory cascades (NOD2, TNFAIP1), gap junction remodeling and cardiac electrical conduction disruption (GJA1/Cx43), cardiac proteostatic defense (HSPB7, BAG3), vascular permeability regulation (VEGFA), and mitochondrial energy metabolism (CPT1A, PRKAB1/AMPK). AMPK signaling was recurrently captured across multiple analytical tiers and pathological dimensions, supporting its role as a central pleiotropic hub—though not the sole pathway—coordinating the inflammation–metabolism intersection in SICM. CELLECT identification of cardiomyocytes as the sole significant cell type localizes the shared genetic liability of SICM-related phenotypes to cardiomyocytes. These findings nominate candidate genes that may inform future investigation of host-directed strategies and risk stratification, pending experimental and clinical validation.

## Figures and Tables

**Table 1:** Genes prioritized by both sCCA-TWAS and FOCUS fine-mapping.

sCCA Feature	Gene	CHR	Heritability Squared	TWAS Z	TWAS FDR	FOCUS PIP	COLOC PP4
sCCA3	*PRKAR2B*	7	2.42 × 10^−1^	4.90781	9.21 × 10^−7^	0.99	0.983
sCCA2	*VTN*	17	9.37 × 10^−2^	−5.45962	4.77 × 10^−8^	0.999	0.164
sCCA1	*TNFAIP1*	17	6.37 × 10^−2^	5.27353	1.34 × 10^−7^	0.996	0.055
sCCA3	*PRKAB1*	12	2.29 × 10^−1^	−5.50895	3.61 × 10^−8^	0.999	0.811
sCCA3	*PLEKHA3*	2	1.10 × 10^−1^	5.19801	2.01 × 10^−7^	0.821	0.046
sCCA2	*HMGN3*	6	0.1991	−7.09877	1.26 × 10^−12^	1	0.972
sCCA1	*PDZRN3*	3	5.14 × 10^−1^	5.982	2.20 × 10^−9^	1	0.999
sCCA2	*PDZRN3*	3	3.17 × 10^−1^	−5.64493	1.65 × 10^−8^	1	0.996
sCCA3	*PDZRN3*	3	3.54 × 10^−1^	5.73526	9.74 × 10^−9^	1	0.999
sCCA1	*PFKFB2*	1	3.46 × 10^−1^	4.900538	9.56 × 10^−7^	0.82	0.987
sCCA1	*CCDC136*	7	2.23 × 10^−1^	6.30976	2.79 × 10^−10^	0.967	0.989
sCCA2	*SLC6A6*	3	1.02 × 10^−1^	6.50605	7.72 × 10^−11^	1	0.986
sCCA1	*SERINC3*	20	5.93 × 10^−1^	−6.65703	2.79 × 10^−11^	1	1
sCCA2	*SERINC3*	20	2.32 × 10^−1^	−6.57091	5.00 × 10^−11^	1	1
sCCA3	*SERINC3*	20	0.3544	−6.7194	1.82 × 10^−11^	1	1
sCCA2	*METTL14*	4	0.2298	5.43352	5.53 × 10^−8^	0.897	0.508
sCCA2	*RASA1*	5	0.1484	4.8903	1.01 × 10^−6^	0.997	0.995
sCCA1	*SEC24D*	4	0.2404	−5.12793	2.93 × 10^−7^	0.958	0.361
sCCA3	*BAG3*	10	0.132	5.113384	3.16 × 10^−7^	1	0.138
sCCA3	*GJA1*	6	0.1232	7.52472	5.28 × 10^−14^	1	0.99
sCCA2	*C7orf31*	7	0.1404	5.22471	1.74 × 10^−7^	1	0.991
sCCA2	*PRKCA*	17	0.3509	5.32607	1.00 × 10^−7^	0.999	0.79
sCCA3	*NSMCE2*	8	0.0924	5.18072	2.21 × 10^−7^	0.892	0.039
sCCA3	*SENP3*	17	0.2415	5.60072	2.13 × 10^−8^	0.98	0.423
sCCA2	*FBLIM1*	1	0.0976	−7.252442	4.09 × 10^−13^	1	0.015
sCCA2	*ALB*	4	0.0661	5.22255	1.76 × 10^−7^	0.942	0.958
sCCA2	*MTSS1*	8	0.2836	−9.529685	1.58 × 10^−21^	1	0.104
sCCA1	*HSPB7*	1	0.4337	8.947941	3.62 × 10^−19^	1	0.992
sCCA3	*HSPB7*	1	0.2683	−9.70122	2.98 × 10^−22^	1	0.995
sCCA1	*SLC35G5*	8	0.2275	6.36356	1.97 × 10^−10^	0.95	0.021
sCCA2	*SLC35G5*	8	0.2224	6.363564	1.97 × 10^−10^	0.991	0.021
sCCA3	*SLC35G5*	8	0.227	−5.44816	5.09 × 10^−8^	0.959	0.008
sCCA2	*PRKRA*	2	0.0427	7.201726	5.95 × 10^−13^	1	0.896
sCCA3	*FAM167B*	1	0.0866	−6.69921	2.10 × 10^−11^	1	0.787
sCCA2	*MCMBP*	10	0.1067	−6.523147	6.88 × 10^−11^	1	0.792
sCCA2	*LIME1*	20	0.2196	5.08037	3.77 × 10^−7^	0.976	0.993
sCCA1	*AFG3L1P*	16	0.3171	−7.435347	1.04 × 10^−13^	1	1
sCCA2	*AFG3L1P*	16	0.1951	7.31	2.72 × 10^−13^	1	1
sCCA3	*AFG3L1P*	16	0.181	−6.21409	5.16 × 10^−10^	1	1
sCCA1	*TTN-AS1*	2	0.0727	−7.34	2.10 × 10^−13^	1	0.035
sCCA2	*TNFSF12*	17	0.2803	5.7283	1.01 × 10^−8^	0.803	0.671
sCCA2	*CTD-2260A17.2*	5	0.1536	−5.65607	1.55 × 10^−8^	1	0.834
sCCA1	*FOXN3-AS1*	14	0.1998	4.99199	5.98 × 10^−7^	0.997	0.985
sCCA3	*MILR1*	17	0.3668	−4.84808	1.25 × 10^−6^	0.924	0.11
sCCA2	*RP11-155G14.5*	7	0.1444	−5.59203	2.24 × 10^−8^	0.82	0.918
sCCA1	*CTA-212A2.1*	22	0.0956	5.17162	2.32 × 10^−7^	0.998	0.506
sCCA2	*ERICD*	8	0.102	−4.956945	7.16 × 10^−7^	0.966	0.869
sCCA2	*RBM5-AS1*	3	0.0755	−5.21121	1.88 × 10^−7^	0.865	0.936

Notes: Genes appearing in more than one row represent associations detected through different cross-tissue canonical features (sCCA1–sCCA3) within the sCCA-TWAS framework, not duplicate records.

## Data Availability

The data presented in this study are available in the GWAS Catalog at https://www.ebi.ac.uk/gwas/ (accessed on 25 March 2026) (reference numbers GCST90797572, GCST90019013, GCST90479489, and GCST90268120); and IEU OpenGWAS at https://gwas.mrcieu.ac.uk/ (accessed on 25 March 2026) (reference numbers ebi-a-GCST90018806 and ieu-b-4980).

## Supplementary Materials

The following supporting information can be downloaded at https://www.mdpi.com/article/10.3390/genes17070751/s1: Figure S1. Quantile–quantile plot of the multivariate GWAS association statistics for the SICM-related latent factor; Table S1: Summary of GWAS data sources for six SICM-related phenotypes; Table S2: SNP-based heritability estimates for the six SICM-related traits; Table S3: Pairwise genetic correlations among the six SICM-related traits; Table S4: Goodness-of-fit indices for the confirmatory one-factor Genomic SEM model; Table S5: Standardized factor loadings and residual variances for the latent SICM factor; Table S6: Genome-wide significant SNPs identified by multivariate GWAS of the latent SICM factor; Table S7: Independent lead SNPs after LD-based clumping; Table S8: Novelty assessment of lead SNPs relative to constituent single-trait GWAS; Table S9: Prior trait associations of lead variants cataloged in the GWAS literature; Table S10: Bayesian fine-mapping results across genome-wide significant loci; Table S11: MAGMA gene-level association results for the latent SICM factor; Table S12: Gene-set enrichment analysis results from MSigDB reference collections; Table S13: CELLECT cell-type heritability enrichment results; Table S14: Spatial transcriptomic mapping results from gsMap at E16.5; Table S15: Stratified LD score regression results across functional genomic annotations.

## Abbreviations

The following abbreviations are used in this manuscript:

SICM	Sepsis-induced cardiomyopathy
GWAS	Genome-wide association study
HF	Heart failure
TLR	Toll-like receptor
VEGFR-1	Vascular endothelial growth factor receptor 1
MR	Mendelian randomization
SEM	Structural equation modeling
cTnI	Circulating cardiac troponin I
LVEF	Left ventricular ejection fraction
SNPs	Single-nucleotide polymorphisms
MAF	Minor allele frequency
MHC	Major histocompatibility complex
LDSC	Linkage disequilibrium score regression
CFI	Comparative fit index
SRMR	Standardized root mean square residual
χ^2^	Chi-square
AIC	Akaike information criterion
FUMA	Functional Mapping and Annotation
PP	Posterior probability
sCCA-TWAS	Sparse canonical correlation analysis-based transcriptome-wide association study
eQTL	Expression quantitative trait locus
GTEx	Genotype-Tissue Expression
FOCUS	Fine-mapping of CaUsal gene Sets
PIP	Posterior inclusion probability
GSEA	Gene-set enrichment analysis
MSigDB	Molecular Signatures Database
scRNA-seq	Single-cell RNA sequencing
S-LDSC	Stratified LDSC
gsMaP	Genetically informed spatial mapping
MOSTA	Mouse embryonic spatial transcriptomics data
