# Supplementary figures and images for: Genomic Structural Equation Modeling Reveals Shared Genetic Architecture and Pleiotropic Hub Genes of Sepsis-Induced Cardiomyopathy
Source: Genes (Basel). 2026 Jun 30;17(7):751. doi: 10.3390/genes17070751 (PMC13408642; doi:10.3390/genes17070751)

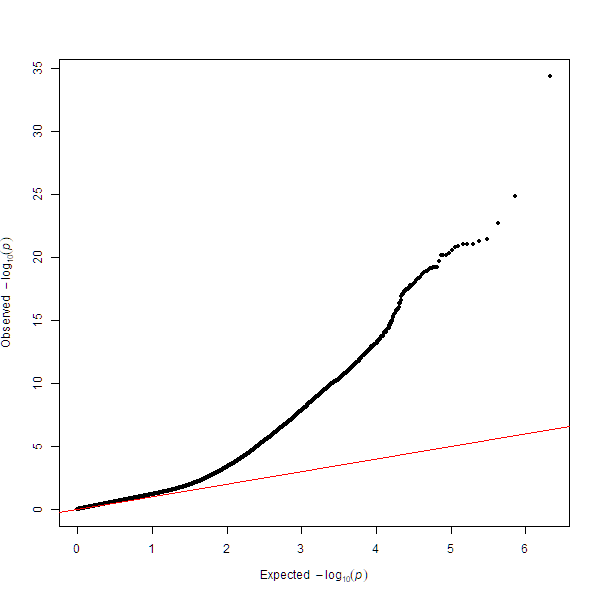

Supplement: Supplementary file 1 [file genes-17-00751-s001.zip › Figure S1.png]
